# The identification of high-performing antibodies for transmembrane protein 106B (TMEM106B) for use in Western blot, immunoprecipitation, and immunofluorescence

**DOI:** 10.12688/f1000research.131333.1

**Published:** 2023-03-21

**Authors:** Riham Ayoubi, Maryam Fotouhi, Kathleen Southern, Ritika Bhajiawala, Rebeka Fanti, Panagiotis Prinos, Peter S. McPherson, Carl Laflamme, NeuroSGC/YCharOS/EDDU collaborative group, ABIF Consortium

**Affiliations:** 1 Department of Neurology and Neurosurgery, Structural Genomics Consortium, The Montreal Neurological Institute, McGill University, Montreal, Quebec, H3A 2B4, Canada; 2 Structural Genomics Consortium, University of Toronto, Toronto, Quebec, M5G 1L7, Canada

**Keywords:** Uniprot ID Q9NUM4, TMEM106B, Transmembrane protein 106B, antibody characterization, antibody validation, Western Blot, immunoprecipitation, immunofluorescence

## Abstract

Transmembrane protein 106B (TMEM106B), a protein that is localized to the lysosome, is genetically linked to many neurodegenerative diseases and forms fibrils in diseased brains. The reproducibility of TMEM106B research would be enhanced if the community had access to well-characterized anti-TMEM106B antibodies. In this study, we characterized six commercially available TMEM106B antibodies for their performance in Western blot, immunoprecipitation, and immunofluorescence, using a standardized experimental protocol based on comparing read-outs in knockout cell lines and isogenic parental controls. We identified many high-performing antibodies and encourage readers to use this report as a guide to select the most appropriate antibody for their specific needs.

## Introduction

Transmembrane protein 106B (TMEM106B) is a genetic risk variant for many neurodegenerative diseases. The presence of the *TMEM106B* major risk allele, rs1990622, is suspected to be a risk factor and disease modifier for Frontotemporal Dementia (FTD), with few studies investigating its potential role in Amyotrophic Lateral Sclerosis (ALS) pathogenesis. ^1^^–^^5^

TMEM106B is a transmembrane endosomal and lysosomal glycoprotein. The protein has garnered interest lately, with the discovery that a 135 amino acid portion of the protein from its luminal C-terminal domain forms fibrils in the brains of patients with frontotemporal lobar degeneration, progressive supranuclear palsy, and dementia with Lewy bodies. ^6^^,^^7^

The roles of TMEM106B fibrils in normal lysosomal function or disease pathogenesis are not known, nor is the mechanism by which the protein is proteolyzed, or forms fibrils. ^7^ Mechanistic studies would be greatly facilitated with the availability of high-quality validated antibodies. Here, we compared the performance of a range of commercially available antibodies for TMEM106B and characterized several high-quality antibodies for Western blot, immunoprecipitation and immunofluorescence, enabling biochemical and cellular assessment of TMEM106B properties and function.

## Results and discussion

Our standard protocol involved comparing readouts from wild-type (WT) and knockout (KO) cells. ^8^^,^^9^ The first step was to identify a cell line(s) that expresses sufficient levels of TMEM106B to generate a measurable signal. To this end, we examined the DepMap transcriptomics databases to identify all cell lines that express the target at levels greater than 2.5 log _2_ (transcripts per million “TPM” +1), which we have found to be a suitable cut-off (Cancer Dependency Map Portal, RRID:SCR_017655). Commercially available HAP1 cells expressed the *TMEM106* *B* at RNA levels above the average range of cancer cells analyzed. The parental and KO HAP1 cell lines were obtained from Horizon Discovery ( Table 1).

**Table 1.** Summary of the cell lines used.

Institution	Catalog number	RRID (Cellosaurus)	Cell line	Genotype
Horizon Discovery	C631	CVCL_Y019	HAP1	WT
Horizon Discovery	HZGHC005877c010	CVCL_XU38	HAP1	*TMEM106B* KO

**Figure 1.**
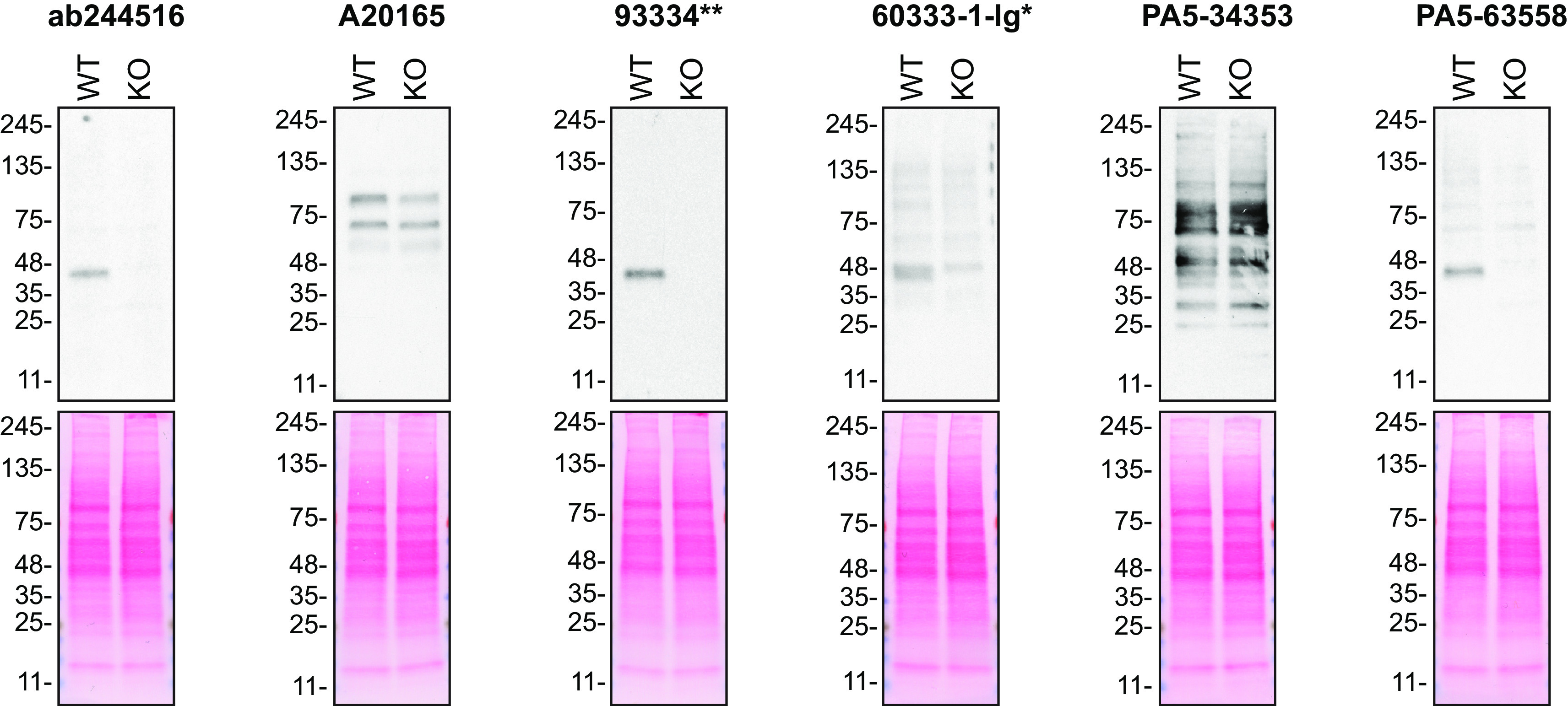
Transmembrane protein 106B antibody screening by Western Blot. Lysates of HAP1 (WT and *TMEM106B* KO) were prepared and 15 μg of protein were processed for Western Blot with the indicated TMEM106B antibodies. The Ponceau stained transfers of each blot are presented are shown equal loading of WT and KO lysates and protein transfer efficiency from the acrylamide gels to the nitrocellulose membrane. Antibody dilutions were chosen according to the recommendations of the antibody supplier. Exceptions were given for antibodies 93334** and 60333-1-Ig*, which were titrated to 1/500 and 1/2000, respectively, as the signal was too weak when following the supplier’s recommendations. Antibody dilution used: ab244516 at 1/500, A20165 at 1/2000, 93334** at 1/500, 60333-1-lg* at 1/2000, PA5-34353 at 1/500, and PA5-63558 at 1/200.Predicted band size: 31 kDa. *= monoclonal antibody, **= recombinant antibody.

For Western blot experiments, we resolved proteins from WT and *TMEM106B* KO cell extracts and probed them side-by-side with all antibodies in parallel ( Figure 1).

**Figure 2.**
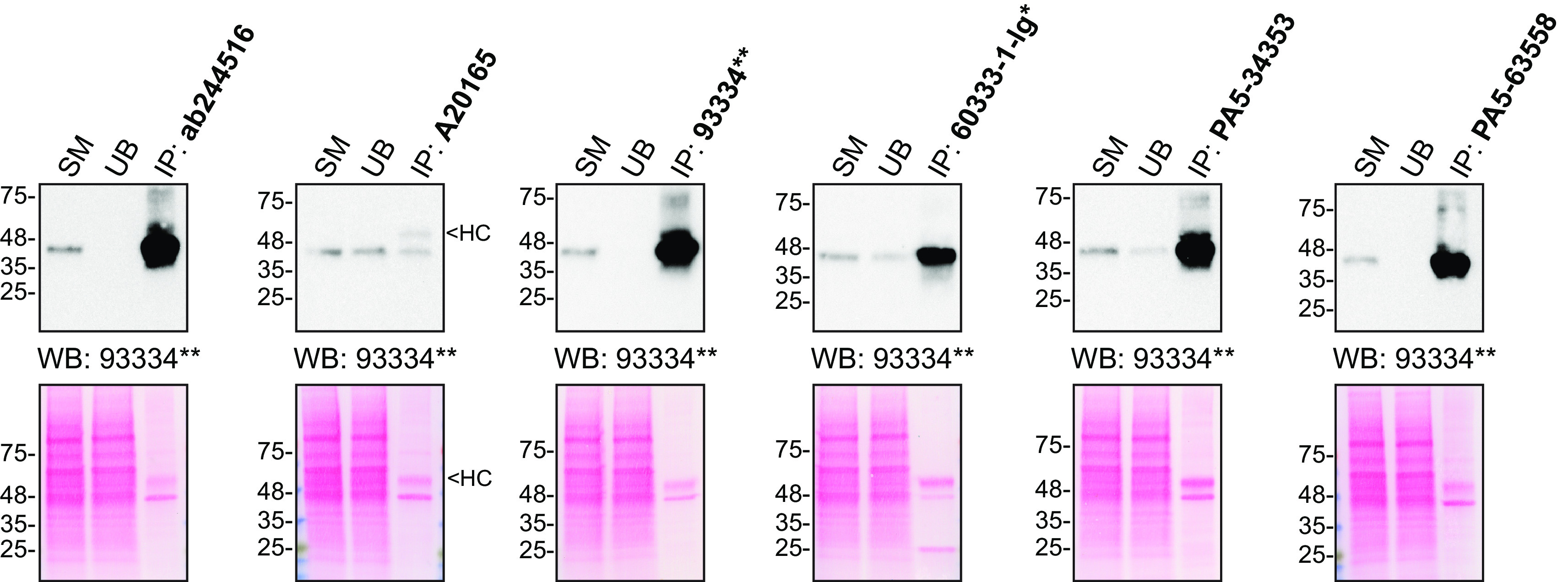
Transmembrane protein 106B antibody screening by immunoprecipitation. HAP1 lysates were prepared, and IP was performed using 2.0 μg of the indicated TMEM106B antibodies pre-coupled to Dynabeads protein G or protein A. Samples were washed and processed for Western Blot with the indicated TMEM106B antibody. For Western Blot, 93334** was used at 1/500. The Ponceau stained transfers of each blot are shown for similar reasons as in Figure 1. SM=2% starting material; UB=2% unbound fraction; IP=immunoprecipitate; HC=antibody heavy chain. *= monoclonal antibody, **= recombinant antibody.

For immunoprecipitation experiments, we used the antibodies to immunopurify TMEM106B from HAP1 cell extracts. The performance of each antibody was evaluated by detecting the TMEM106B protein in extracts, in the immunodepleted extracts and in the immunoprecipitates ( Figure 2).

**Figure 3.**
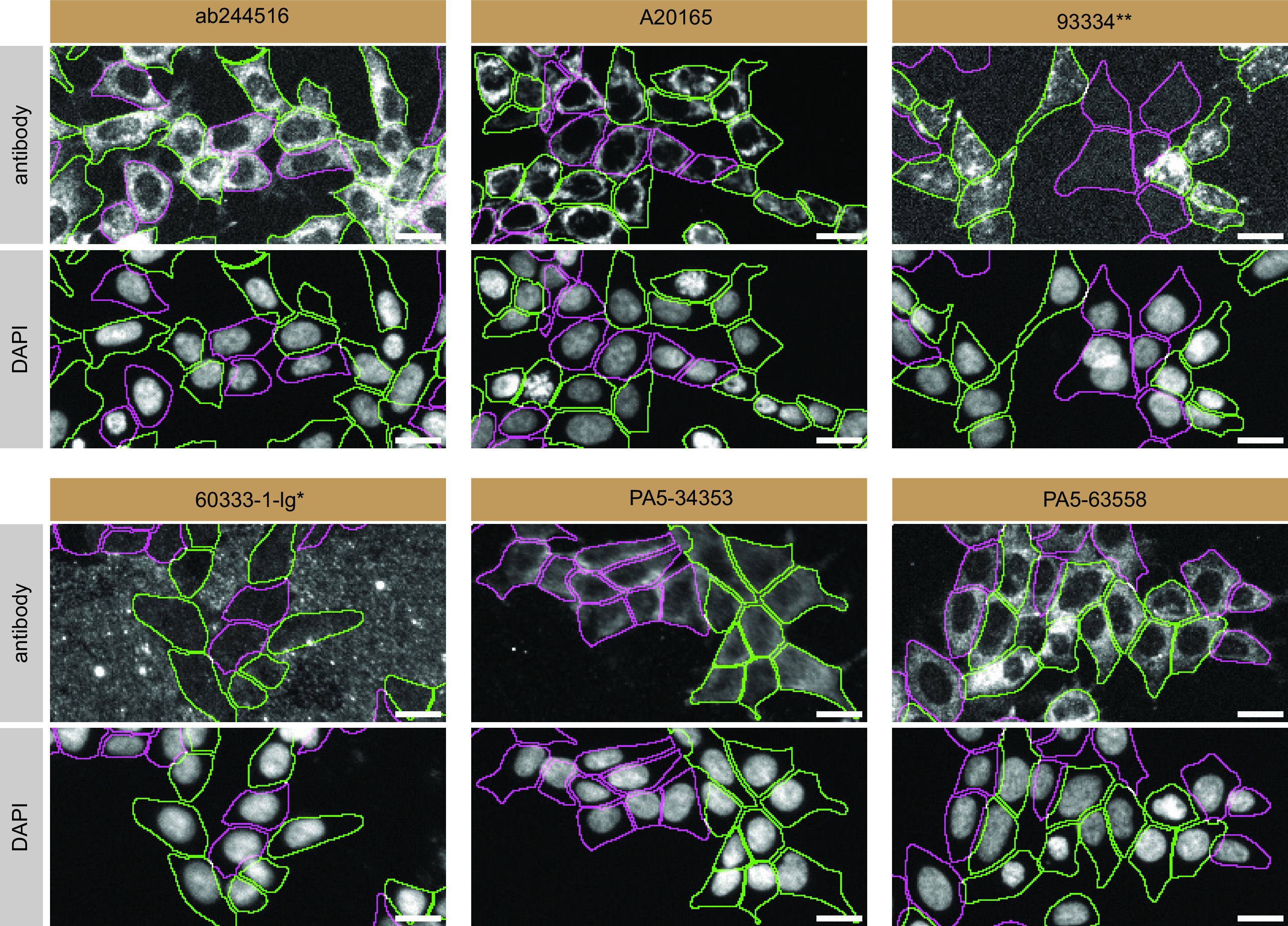
Transmembrane protein 106B antibody screening by immunofluorescence. HAP1 WT and *TMEM106B* KO cells were labelled with a green or a far-red fluorescent dye, respectively. WT and KO cells were mixed and plated to a 1:1 ratio in a 96-well plate with a glass bottom. Cells were stained with the indicated TMEM106B antibodies and with the corresponding Alexa-fluor 555 coupled secondary antibody including DAPI. Acquisition of the blue (nucleus-DAPI), green (identification of WT cells), red (antibody staining) and far-red (identification of KO cells) channels were performed. Representative images of the merged blue and red (grayscale) channels are shown. WT and KO cells are outlined with green and magenta dashed line, respectively. When the concentration was not indicated by the supplier, we tested antibodies at 1/1000 or 1/2000. At this concentration, the signal from each antibody was in the range of detection of the microscope used. Antibody dilution used: ab244516 at 1/100, A20165 at 1/2000, 93334** at 1/100, 60333-1-lg* at 1/2000, PA5-34353 at 1/1000, and PA5-63558 at 1/100. Bars = 10 μm. *= monoclonal antibody, **= recombinant antibody.

For immunofluorescence, as described previously, antibodies were screened using a mosaic strategy. ^10^ In brief, we plated WT and KO cells together in the same well and imaged both cell types in the same field of view to reduce staining, imaging and image analysis bias ( Figure 3).

In conclusion, we have screened many TMEM106B commercial antibodies by Western blot, immunoprecipitation and immunofluorescence and identified several high-quality antibodies under our standardized experimental conditions.

## Methods

### Antibodies

All TMEM106B antibodies are listed in Table 2 together with their corresponding Research Resource Identifiers, or RRID, to ensure the antibodies are cited properly. ^11^ Peroxidase-conjugated goat anti-mouse and anti-rabbit antibodies are from Thermo Fisher Scientific (cat. number 62-6520 and 65-6120). Alexa-555 conjugated secondary goat anti-rabbit and anti-mouse antibodies are from Thermo Fisher Scientific (cat. number A21429 and A21424)

**Table 2.** Summary of the Transmembrane protein 106B antibodies tested.

Company	Catalog number	Lot number	RRID (Antibody Registry)	Clonality	Clone ID	Host	Concentration (μg/μl)	Vendors recommended applications
Abcam	ab244516	GR3421643	AB_2924268 ^1^	polyclonal	-	rabbit	0.10	IF
ABclonal	A20165	131370201	AB_2862952	polyclonal	-	rabbit	1.88	Wb
Cell Signaling Technology	93334 **	1	AB_2924267 ^1^	recombinant-mono	E7H7Z	rabbit	0.10	Wb, IP, IF
Proteintech	60333-1-lg *	10002132	AB_2881442	monoclonal	5D1F8	mouse	2.00	Wb
Thermo Fisher Scientific	PA5-34353	XD3572518	AB_2551705	polyclonal	-	rabbit	1.00	Wb
Thermo Fisher Scientific	PA5-63558	XD3571635	AB_2648556	polyclonal	-	rabbit	0.10	IF

Wb = Western blot, IF = immunofluorescence, IP = immunoprecipitation.

* Monoclonal antibody.

** Recombinant antibody.

^1^ Refer to RRID recently added to the Antibody Registry (on January 2023), they will be available on the Registry website in coming weeks.

### Cell culture

Both HAP1 WT and *TMEM106B* KO cell lines used are listed in Table 1, together with their corresponding RRID, to ensure the cell lines are cited properly. ^12^ Cells were cultured in DMEM high glucose (GE Healthcare, cat. number SH30081.01) containing 10% fetal bovine serum (Wisent, cat. number 080450), 2 mM L-glutamate (Wisent, cat. number 609065), 100 IU penicillin and 100 μg/mL streptomycin (Wisent, cat. number 450201).

### Antibody screening by Western blot

Western blot experiments were performed as described in our standard operating procedure. ^13^ HAP1 WT and *TMEM106B* KO were collected in RIPA buffer (25mM Tris-HCl pH 7.6, 150mM NaCl, 1% NP-40, 1% sodium deoxycholate, 0.1% SDS) from Thermo Fisher Scientific (cat number 0089901), supplemented with 1x protease inhibitor cocktail mix from MilliporeSigma (cat. number 78429). Lysates were sonicated briefly and incubated for 30 min on ice. Lysates were spun at ~110,000 x g for 15 min at 4°C and equal protein aliquots of the supernatants were analyzed by SDS-PAGE and Western Blot. BLUelf prestained protein ladder from GeneDireX (cat. number PM008-0500) was used.

Western blots were performed with pre-cast mini 4-15% gradient polyacrylamide gels from Bio-Rad (cat. number 4561084) and transferred on nitrocellulose membranes. Proteins on the blots were visualized with Ponceau staining which is scanned to show together with individual Western blots. Blots were blocked with 5% milk for 1 h, and antibodies were incubated overnight at 4°C with 5% bovine serum albumin (BSA) (Wisent, cat. number 800-095) in TBS with 0,1% Tween 20 (TBST) (Cell Signaling Technology, cat. number 9997). Following three washes with TBST, the peroxidase conjugated secondary antibody was incubated at a dilution of ~0.2 μg/mL in TBST with 5% milk for 1 hr at room temperature followed by three washes with TBST. Membranes are incubated with Pierce ECL from Thermo Fisher Scientific (cat. number 32106) prior to detection with HyBlot CL autoradiography films from Denville (cat. number 1159T41).

### Antibody screening by immunoprecipitation

Immunoprecipitation was performed as described in our standard operating procedure. ^14^ Antibody-bead conjugates were prepared by adding 2 μg of antibody to 500 μL of Pierce IP Lysis Buffer from Thermo Fisher Scientific (cat. number 87788) in a 1.5 mL microcentrifuge tube together with 30 μL of Dynabeads protein A- (for rabbit antibodies) or protein G- (for mouse antibodies) from Thermo Fisher Scientific (cat. number 10002D and 10004D, respectively). Tubes were rocked ~2 hours at 4°C followed by several washes to remove unbound antibodies.

HAP1 WT were collected in Pierce IP buffer (25 mM Tris-HCl pH 7.4, 150 mM NaCl, 1 mM EDTA, 1% NP-40 and 5% glycerol) from Thermo Fisher Scientific (cat. number 87788), supplemented with protease inhibitor from MilliporeSigma (cat. number P8340). Lysates were rocked for 30 min at 4°C and spun at 110,000 x g for 15 min at 4°C. 0.5 mL aliquots at 2.0 mg/mL of lysate were incubated with an antibody-bead conjugate for ~2 hrs at 4°C. The unbound fractions were collected, and beads were subsequently washed three times with 1.0 mL of IP lysis buffer and processed for SDS-PAGE and Western blot on a pre-cast mini 4-15% polyacrylamide gel. Prot-A:HRP (MilliporeSigma, cat. number P8651) was used as a secondary detection system at a dilution of 0.4 μg/mL.

### Antibody screening by immunofluorescence

Immunofluorescence was performed as described in our standard operating procedure. ^10^ HAP1 WT and *TMEM106B* KO were labelled with CellTracker green (Thermo Fisher Scientific, cat. number C2925) or CellTracker deep red (Thermo Fisher Scientific, cat. number C34565) fluorescence dye, respectively. The nuclei were labelled with DAPI (Thermo Fisher Scientific, cat. number D3571) fluorescent stain. WT and KO cells were plated in 96 well glass plates (Perkin Elmer, cat. number 6055300) as a mosaic and incubated for 24 hrs in a cell culture incubator at 37 ^o^C, 5% CO _2_. Cells were fixed in 4% paraformaldehyde (PFA) (Beantown chemical, cat. number 140770-10mL) in phosphate buffered saline (PBS) (Wisent, cat. number 311-010-CL) for 15 min at room temperature and then washed three times with PBS. Cells were permeabilized in PBS with 0.1% Triton X-100 (Thermo Fisher Scientific, cat. number BP151-500) for 10 min at room temperature and blocked with PBS containing 5% BSA, 5% goat serum (Gibco, cat. number 16210-064) and 0.01% Triton X-100 for 30 min at room temperature. Cells were incubated with IF buffer (PBS, 5% BSA, 0.01% Triton X-100) containing the primary TMEM106B antibodies overnight at 4°C. Cells were then washed 3 × 10 min with IF buffer and incubated with the corresponding Alexa Fluor 555-conjugated secondary antibodies in IF buffer at a dilution of 1.0 μg/mL for 1 hr at room temperature with DAPI. Cells were washed 3 × 10 min with IF buffer and once with PBS.

Images were acquired on an ImageXpress micro widefield high-content microscopy system (Molecular Devices), using a 20x/0.45 NA air objective lens and scientific CMOS camera (16-bit, 1.97mm field of view), equipped with 395, 475, 555 and 635 nm solid state LED lights (Lumencor Aura III light engine) and bandpass emission filters (432/36 nm, 520/35 nm, 600/37 nm and 692/40 nm) to excite and capture fluorescence emission for DAPI, CellTracker Green, Alexa fluor 555 and CellTracker Red, respectively. Images had pixel sizes of 0.68 × 0.68 microns. Exposure time was set with maximal (relevant) pixel intensity ~80% of dynamic range and verified on multiple wells before acquisition. Since the IF staining varied depending on the primary antibody used, the exposure time was set using the most intensely stained well as reference. Frequently, the focal plane varied slightly within a single field of view. To remedy this issue, a stack of three images per channel was acquired at a z-interval of 4 microns per field and best focus projections were generated during the acquisition (MetaXpress v6.7.1, Molecular Devices). Segmentation was carried out on the projections of CellTracker channels using CellPose v1.0 on green (WT) and far-red (KO) channels, using as parameters the ‘cyto’ model to detect whole cells, and using an estimated diameter tested for each cell type, between 15 and 20 microns. ^15^ Masks were used to generate cell outlines for intensity quantification. Figures were assembled with Adobe Illustrator.

## Data Availability

Zenodo: Antibody Characterization Report for Transmembrane protein 106B, https://doi.org/10.5281/zenodo.7459629. ^16^ Zenodo: Dataset for the Transmembrane protein 106B antibody screening study, https://doi.org/10.5281/zenodo.7587647. ^17^ Data are available under the terms of the Creative Commons Attribution 4.0 International license (CC-BY 4.0).
