# Supplementary material for: The identification of high-performing antibodies for transmembrane protein 106B (TMEM106B) for use in Western blot, immunoprecipitation, and immunofluorescence
Source: F1000Res. 2023 Mar 21;12:308. [Version 1] doi: 10.12688/f1000research.131333.1 (PMC10403746; doi:10.12688/f1000research.131333.1)
Supplement: Peer review history

# Peer review: Reviewer response for version 1

**DOI:** 10.5256/f1000research.144167.r178524

**Published:** 2023-08-04

**Authors:** Giuseppe Legname

**Affiliations:** 1 Laboratory of Prion Biology, Department of Neuroscience, Scuola Internazionale Superiore di Studi Avanzati, Trieste, Friuli-Venezia Giulia, Italy

This manuscript describes the various antibodies available for the study of TMEM106B. This is a useful methodological paper. The work is well organized, and the methodologies appropriately presented. I would like to suggest comparing the methods and results with those suggested by the vendors and discuss advantages to use the methodologies presented by the authors.

Are sufficient details of methods and materials provided to allow replication by others?

Yes

Is the rationale for creating the dataset(s) clearly described?

Yes

Are the datasets clearly presented in a useable and accessible format?

Yes

Are the protocols appropriate and is the work technically sound?

Yes

Reviewer Expertise:

NA

I confirm that I have read this submission and believe that I have an appropriate level of expertise to confirm that it is of an acceptable scientific standard, however I have significant reservations, as outlined above.

# Peer review: Reviewer response for version 1

**DOI:** 10.5256/f1000research.144167.r183540

**Published:** 2023-08-04

**Authors:** Christian Peter Moritz

**Affiliations:** 1 Department of Neurology, University hospital of Saint-Etienne, Saint-Etienne, France; 2 Synaptopathies and autoantibodies (SynatAc) team, Mechanisms In Integrated Life Sciences (MELIS) Laboratory, Institute NeuroMyoGène (INMG), INSERM U1314/CNRS UMR 5284, Universités de Lyon, Université Claude Bernard Lyon 1, Lyon, France; 3 University Jean Monnet, Saint-Etienne, France

In their article, Ayoubi and colleagues are reporting about their quality tests of six different commercial anti-TMEM106B antibodies using Western blotting, immunoprecipitation, and immunocytochemistry. 

 The article is interesting and useful for groups working with this protein.

 Doing KO controls to test antibodies is considered the gold standard of validation tests. Nevertheless, unfortunately it is not done very often. That’s why I appreciate the work of these authors.

 I would consider the following concerns.

**MAJOR CONCERNS:**

1) Chapter: “Results and discussion”:
  1. In my experience it is common to describe the results in the result chapter instead of just referring to the figures.
  2. There is no discussion in this chapter yet. The authors should interpret their results in a more concrete way. Which antibodies do they recommend for which method?
    Interesting to mention in my eyes: 2 out of 6 tested antibodies don’t work in Western blotting, as they don’t bind the protein of interest, but they nonspecifically bind other proteins. One further antibody (60333-1-lg) nonspecifically binds other proteins in addition to binding the protein of interest.
    In total, half of the antibodies show unspecific binding, which could harm result interpretation when using this antibody without having a KO control.
Article type "Data Note": In my perspective this article is not a "data note" in the classical sense (Journal info: "Data Notes are brief descriptions of datasets that promote the potential reuse of research data and include details of why and how the data were created; they do not include any analyses or conclusions").
  The authors do not present a classical data set however. To me, an analysis and conclusions are necessary. Hence, I would ask the authors to consider another article type. Is there something like a "Technical note"?

**MINOR CONCERNS:**

1. In their introduction, the authors speak of “a 135 amino acid portion of the protein from its luminal C-terminal domain”, that might play a pathophysiological role.
  Given this information, it might be interesting which of the six antibodies have epitopes in that specific domain. I there any information available? Some ideas to address this questions methodologically ?
2. Results and discussion: “For Western blot experiments, we resolved proteins from WT and TMEM106B KO cell extracts and probed them side-by side with all antibodies in parallel (Figure 1).”
  What means “side-by side” here? Do they come from the same membrane in order to avoid membrane-to-membrane variation? In the best case, in future experiments, the authors could even considering cutting the lanes vertically in order to even avoid lane-to-lane variation (cf. Pubmed ID 30768763).
3. Figure 1, panel PA5-34353:
  I am a bit surprised that the “scratches” and inhomogenities in protein transfer is not equally visible in the total protein staining (ponceau). I would kindly ask the authors to check in their data files that the ponceau staining is really originating from the same membrane, because the loading control would be invalid otherwise. 
4. Figure 2: Why is “HC” (antibody heavy chain) only shown in the second panel? I would recommend showing it in the first panel only, or in all panels.
5. Figure 2: I do not understand why there is written “WB: 93334**” below each of the 6 Western blots.
6. Figure 3: Is the Alexa-fluor 555 not interfering with the fluorescence stainings that were applied to distinguish WT from KO?
7. Concluding sentence in the results section: “we have screened many TMEM106B commercial antibodies”
  I recommend avoiding vague words like “many” where a more concrete indication (“six”) is possible. Same for the word “several” in the same sentence.

Are sufficient details of methods and materials provided to allow replication by others?

Yes

Is the rationale for creating the dataset(s) clearly described?

Yes

Are the datasets clearly presented in a useable and accessible format?

Yes

Are the protocols appropriate and is the work technically sound?

Yes

Reviewer Expertise:

Western blotting, antibodies, autoantibodies, ELISA, method development

I confirm that I have read this submission and believe that I have an appropriate level of expertise to confirm that it is of an acceptable scientific standard, however I have significant reservations, as outlined above.

# Peer review: Reviewer response for version 1

**DOI:** 10.5256/f1000research.144167.r173851

**Published:** 2023-06-02

**Authors:** Dawn Smallwood

**Affiliations:** 1 School of Allied Health Sciences, De Montfort University, Leicester, England, UK

This is a useful paper, highlighting the need for antibody validation. There is important information here for TMEM106B antibody users and clear methodology for those wishing to test antibodies in their own area. Experiments are well carried out and well controlled, and figures are clear. The lack of results analysis means that figures can be interpreted by the reader for their own experimental needs, however, any misinterpretation by a reader could be avoided by adding a column to Table 2 showing the study findings alongside ‘Vendors recommended applications’.

 The number of technical replicates for each method should be stated.

 Full blots could be shown for immunoprecipitation results (Figure 2).

 I would have found figures easier to understand with consistent naming of HAP1 WT and KO cells as HAP1 WT and HAP1 KO rather than a combination of WT and THEM106B KO (Figure 1), HAP1 (Figure 2) and WT and KO (Figure 3).

 Units could be added to the markers in Figures 1 and 2.

Are sufficient details of methods and materials provided to allow replication by others?

Yes

Is the rationale for creating the dataset(s) clearly described?

Yes

Are the datasets clearly presented in a useable and accessible format?

Yes

Are the protocols appropriate and is the work technically sound?

Yes

Reviewer Expertise:

Molecular biology, cell culture, medical genetics

I confirm that I have read this submission and believe that I have an appropriate level of expertise to confirm that it is of an acceptable scientific standard.
